# Ultraperformance liquid chromatography-quadrupole time-of-flight mass spectrometry based untargeted metabolomics to reveal the characteristics of *Dictyophora rubrovolvata* from different drying methods

**DOI:** 10.3389/fnut.2022.1056598

**Published:** 2022-11-28

**Authors:** Hui Dong, Changyan Zhou, Xiaobei Li, Haotian Gu, Hengchao E, Yanmei Zhang, Feng Zhou, Zhiyong Zhao, Tingting Fan, Huan Lu, Min Cai, Xiaoyan Zhao

**Affiliations:** ^1^Laboratory of Agro-Food Quality and Safety Risk Assessment (Shanghai), Institute of Agro-Food Quality Standard and Testing Technology, Shanghai Academy of Agricultural Sciences, Shanghai, China; ^2^Shanghai Engineering Research Center of Low-Carbon Agriculture (SERCLA), Eco-environmental Protection Research Institute, Shanghai Academy of Agricultural Science, Shanghai, China; ^3^National Research Center of Edible Fungi Biotechnology and Engineering, Institute of Edible Fungi, Shanghai Academy of Agricultural Sciences, Shanghai, China

**Keywords:** *Dictyophora rubrovolvata*, metabolomics, drying methods, UPLC-Q-TOF-MS, KEGG, quality evaluation

## Abstract

*Dictyophora rubrovolvata* is a highly valuable and economically important edible fungus whose nutrition and flavor components may vary based on drying methods. Herein, an untargeted ultraperformance liquid chromatography-quadrupole time-of-flight mass spectrometry (UPLC-Q-TOF/MS) metabolomics method combined with multivariate analysis was first performed to characterize the metabolomics profiles of *D. rubrovolvata* upon different drying treatments, viz., coal burning drying (CD), electrothermal hot air drying (ED), and freeze drying (FD). The results indicated that 69 differential metabolites were identified, vastly involving lipids, amino acids, nucleotides, organic acids, carbohydrates, and their derivatives, of which 13 compounds were confirmed as biomarkers in response to diverse drying treatments. The Kyoto Encyclopedia of Genes and Genomes (KEGG) enrichment analysis illustrated that differential metabolites were significantly assigned to 59, 55, and 60 pathways of CD vs. ED, CD vs. FD, and FD vs. ED groups, respectively, with 9 of the top 20 KEGG pathways shared. Specifically, most of lipids, such as fatty acyls, glycerophospholipids and sphingolipids, achieved the highest levels in *D. rubrovolvata* after the CD treatment. ED method substantially enhanced the contents of sterol lipids, nucleotides, organic acids and carbohydrates, while the levels of amino acids, prenol lipids and glycerolipids were elevated dramatically against the FD treatment. Collectively, this study shed light on metabolomic profiles and proposed biomarkers of *D. rubrovolvata* subjected to multiple drying techniques, which may contribute to quality control and drying efficiency in edible fungi production.

## Introduction

*Dictyophora rubrovolvata*, an edible mushroom from the Dictyophora family, is highly valuable and widely consumed in Asian countries due to its attractive appearance, tasty flavor and high nutritive value. In addition, a wealth of biopharmaceutical ingredients involved in antimicrobial, antioxidant, antitumor and immunomodulatory effects have been well-documented, such as polysaccharides, flavonoids, and terpenoids (1–3). With its tremendous market popularity and commercial value, *D. rubrovolvata* has emerged as one of the most characteristic and booming products in the mushroom industry, especially in Guizhou, Sichuan, and Yunnan provinces of China. It is estimated that over 90% of the total annual output of *D. rubrovolvata* was derived from Zhijin County (Guizhou Province), which was registered as the “Agro-product Geographical Indications” in 2010 (1, 4).

Benefiting from the advancement of artificial cultivation technologies, the output and consumption of *D. rubrovolvata* has soared in recent years. Nevertheless, as a kind of water-rich mushroom, *D. rubrovolvata* fresh fruiting bodies will undergo vigorous aging and autophagy processes during postharvest management and preservation, which shrinks their shelf life and hampers consumption and trade (5). Like other mushrooms, drying remains the most common method for the long-term preservation of *D. rubrovolvata* as well as reducing the water content and promoting the formation of aroma, taste and color (6). Previously, multiple drying methods for mushroom processing have been reported, i.e., hot air drying, natural air drying, freeze drying, microwave drying, vacuum drying, etc. (7). This poses a manufacturing challenge since each method has advantages and disadvantages. For instance, coal burning drying has been practiced in large-scale *D. rubrovolvata* processing for decades due to its simple manipulation and low costs. Electrothermal hot air drying has been extensively employed for drying vegetables, fruits, and natural products, and it is also prevalent in *D. rubrovolvata* drying (8). Freeze drying can minimize degradation of the nutritional value of mushrooms. However, due to its energy consumption, the promotion of this technique is generally a problem (9).

Indeed, the quality of *D. rubrovolvata* reflected by flavor, odor, and nutritional metabolites is the paramount factor affecting consumers’ choices (10). Accumulating evidence has demonstrated that ultraperformance liquid chromatography-quadrupole time-of-flight mass spectrometry (UPLC-Q-TOF-MS) with high resolution, sensitivity and peak reproducibility is a promising and powerful approach to rapidly screen and identify the mushroom metabolites (11). During the course of mushroom drying, the contents of endogenous components are subjected to dynamic changes, notably concentrated nutrients and unique flavor formation (12). To date, however, there is a paucity of information concerning influence of the drying processing method on the nutrition and flavor components of *D. rubrovolvata*.

In this study, UPLC-Q-TOF-MS metabolomics was utilized to identify the differential metabolites of *D. rubrovolvata* upon the three different drying treatments, including coal burning drying (CD), electrothermal hot air drying (ED), and freeze drying (FD). The major objective was to better elucidate the effect of different drying methods on the changes of some flavor/nutrition-related components in the drying process of *D. rubrovolvata*. Our results will provide a valuable reference for the selection of the suitable drying technology to retain certain flavor/nutrition-related components in *D. rubrovolvata*.

## Materials and methods

### Samples and reagents

Twenty-four fresh *D. rubrovolvata* samples were collected in Zhijin County (Bijie City, Guizhou Province, China) in July 2021 and were identified as *D. rubrovolvata* by ITS sequencing by Shanghai Personalbio Technology Co., Ltd. (Shanghai, China). The 24 authentic samples were divided into three groups (*n* = 8, *N* = 3) and processed by CD, ED, and FD methods, respectively. The CD group samples were suspended for coal stove drying for up to 8 h. The ED group were performed in a drying chamber (AOBOTE Drier, Foshan, Guangdong, China) with the drying programs set as follows: 60°C for 2 h; 45°C for 4 h; 50°C for 2 h. The FD group were thoroughly freeze-dried using a vacuum freeze dryer at −50°C under a vacuum of 1–10 mbar for 8 h. The moisture content of each sample was shown in Supplementary Table 1. All dried samples from the three groups were ground into powder (passed through a 425 μm mesh sieve) and then stored at −20°C.

HPLC-grade methanol and acetonitrile were purchased from ANPEL Laboratory Technologies Inc. (Shanghai, China). Formic acid and ammonium acetate (high-purity grade, ≥99.0%) were obtained from Sigma Aldrich Co., Ltd. (Shanghai, China). Ultrapure water (18 mΩ) was purified by a Milli-Q water purification system (Millipore, Milford, MA, USA).

### Sample preparation

The sample preparation for metabolomics analysis was performed according to our previous work (13). Briefly, 0.5 g of the ground powder was mixed with 5.0 mL of 75% methanol/water solution (v/v) and then ultrasonically extracted for 30 min. The mixed solution was then centrifuged at 12,000 rpm for 20 min (4°C) to yield the supernatants, followed by filtering through 0.22 μm microfilters and storing at 4°C for UPLC-Q-TOF-MS analysis. The remaining samples were mixed aliquots and vortexed thoroughly as the quality control sample (QC) to test the stability of UPLC-Q-TOF-MS analysis. Pure water was used as the blank control to verify the cleanliness of the system.

### Ultraperformance liquid chromatography-quadrupole time-of-flight mass spectrometry metabolite analysis

Chromatography separation was carried out using an ACQUITY UPLC system (Waters Corporation, Milford, USA). Mobile phase A was acetonitrile and mobile phase B was 0.1% formic acid in water (v/v). A Waters ACQUITY UPLC BEH C18 column (2.1 × 100 mm, 1.7 μm) was employed for sample separation in both positive and negative modes, and the column temperature was set at 35°C. The sample temperature was 4°C and the injection volume was set 3 μL. The gradient program was as follows: 0–2 min, 5% A; 2–15 min, 5–95% A; 15–17 min, 95% A; 17–17.1 min 95–5% A; 17.1–20 min 5% A.

AB SCIEX Triple TOF 5600 System (AB SCIEX, Framingham, MA, USA) was employed for mass spectrometry analysis. The analytical conditions were set as follows: ion spray voltage: +5,500 V (positive ion mode)/−4,500 V (negative ion mode); curtain gas: 35 psi, source temperature: 550°C. Data acquisition was performed in full scan mode (m/z ranges from 100 to 1,000), and the extracts were analyzed in auto MS/MS mode to obtain MS2 data.

### Data processing and multivariate analysis

Progenesis QI (Waters Corporation, Milford, USA) software was employed for the accurate mass data from automatic MS/MS measurements. The processing parameters were performed according to our previous study (13). All potential metabolites were identified by alignment with public databases (Metlin, Pubchem, and Massbank) and relevant published literature and confirmed based on their retention times and fragmentation patterns. The corresponding relative metabolite contents are presented as chromatographic peak area integrals.

Principal component analysis (PCA) and orthogonal partial least-squares-discriminant analysis (OPLS-DA) were carried out to visualize the metabolic alterations among treated groups and discriminate each group. The variable importance in the projection (VIP) denotes the overall contribution of each variable to the OPLS-DA model, and those variables with VIP > 1 and *p*-value < 0.05 (independent-samples *t*-test) were regarded as significant metabolites. The default 7-round cross validation was applied with 1/seventh of the samples excluded from the mathematical model in each round to guard against overfitting. The screening of different metabolites was visualized in the volcano plot. All the above multivariate analyses were performed in R (version 3.6.3). In addition, Kyoto Encyclopedia of Genes and Genomes (KEGG) enrichment analysis was used to assign differential metabolites to specific metabolic pathways^1^ (14).

## Results and discussion

### Ultraperformance liquid chromatography-quadrupole time-of-flight mass spectrometry-based metabolomics analysis of *Dictyophora rubrovolvata*

**FIGURE 1:**
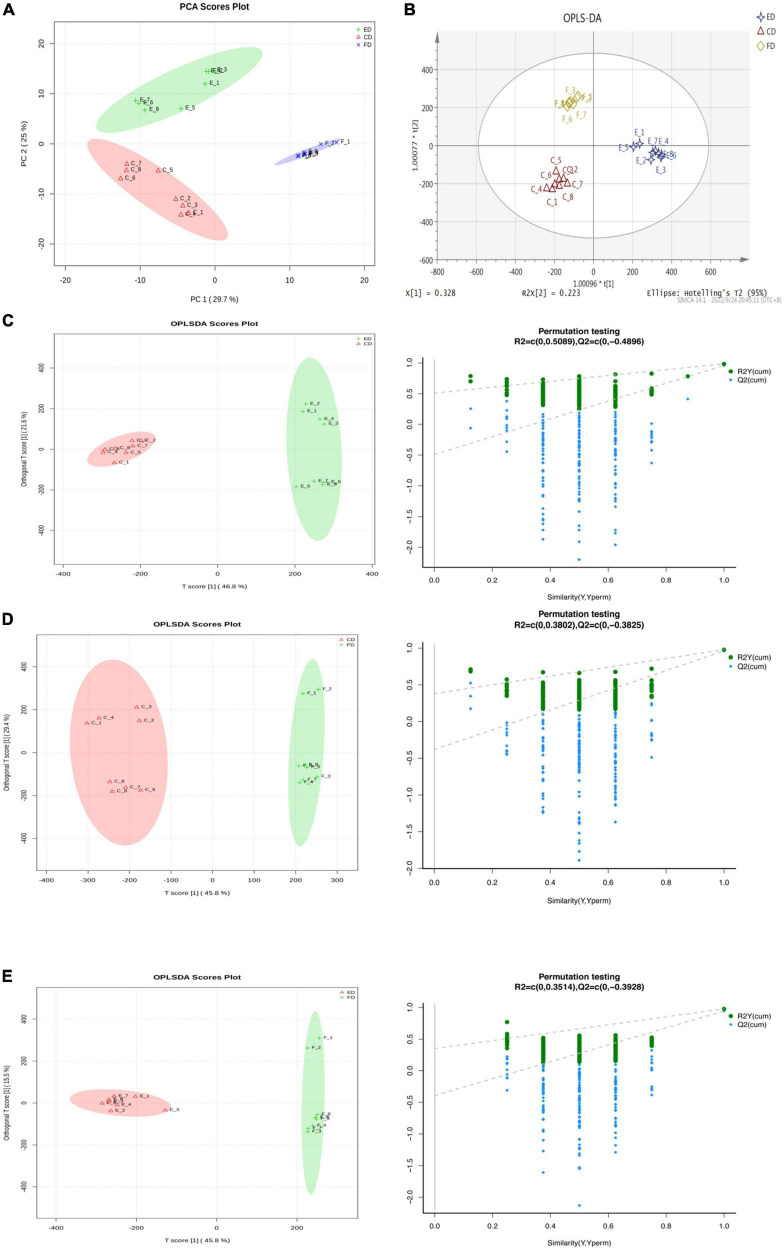
Differential metabolite analysis in *D. rubrovolvata* samples processed by different drying methods. **(A)** PCA score plot of the three drying methods. **(B)** OPLS-DA score plot of the three drying methods. **(C)** OPLS-DA score plot and the permutation tests between CD and ED. **(D)** OPLS-DA score plot and the permutation tests between CD and FD. **(E)** OPLS-DA score plot and the permutation tests between FD and ED.

The metabolites were identified by UPLC-Q-TOF-MS to investigate the chemical composition profile of *D. rubrovolvata* under different drying methods. The total ion chromatograms (TICs) were shown in Supplementary Figure 1 and displayed robust reliability and repeatability of the obtained data. A total of 459 metabolite ions were identified and the number in the positive and negative ion modes was 246 and 213, respectively. After normalization, 318 metabolites (including 140 in the positive ion mode and 178 in the negative mode) were remained and applied for the following statistical analysis. To evaluate whether the metabolite profiles could effectively distinguish the different drying method groups and provide the comparative interpretations, PCA was initially applied to reduce the dimensionality of the dataset and provide an overview of the variation among the groups (15). As shown in the PCA score plot (Figure 1A), the first and second principal components (PC1 and PC2) explained 29.7 and 25% of the accumulative variance contribution rate, respectively. Clear differentiation can be observed corresponding to the drying method in the PCA score plot, indicating that the drying methods can affect the metabolites in *D. rubrovolvata*.

Compared to PCA, OPLS-DA could maximize the separation between the observation groups, hence screening marker compounds were responsible for the differentiation of the three drying methods (Figure 1B) (16). In this study, OPLS-DA with a scatter score plot was applied to compare these samples, and the results are presented in Figures 1C–E. The OPLS-DA models of the processed data showed clearly separated samples, which indicated that the drying method had a strong influence on the composition of metabolites in *D. rubrovolvata*. The model evaluation parameters (R2Y, Q2) obtained after 7 cycles of interactive verification are listed in Table 1. In the S-plot, the R2Y and Q2 were all greater than 0.5, suggesting good predictions of the models and the strong explanatory power of the data (17). For the OPLS-DA permutation test, 200 iterations were performed to determine the effectiveness of the model, and the Q2 of the random model decreased gradually, indicating that the original model was robust. The above results showed that the OPLS-DA model had good predictive performance and could be reliably used to screen differential metabolites.

**TABLE 1:** The detailed comparison of OPLS-DA models and parameters among three drying methods.

Model classes	R2Y (cum)	Q2 (cum)
Coal burning drying vs. Electrothermal hot air drying	0.985	0.958
Coal burning drying vs. Freeze drying	0.978	0.962
Electrothermal hot air drying vs. Freeze drying	0.979	0.945

### Differential metabolite analysis of *Dictyophora rubrovolvata* under different drying methods

**FIGURE 2:**
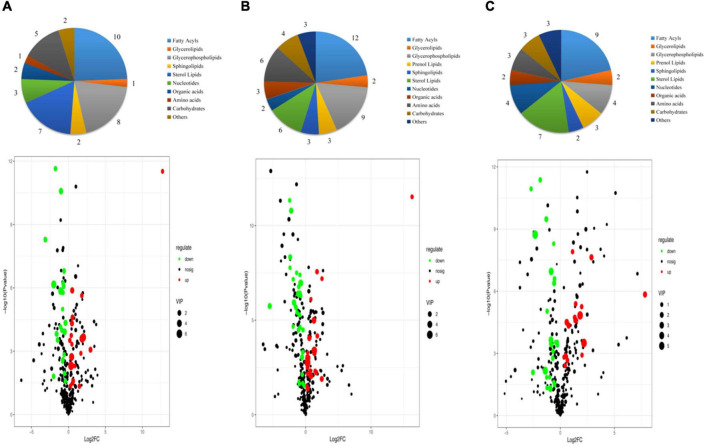
Classification and volcano plot of the differential compounds in different samples: **(A)** CD vs. ED, **(B)** CD vs. FD, **(C)** ED vs. FD.

**FIGURE 3:**
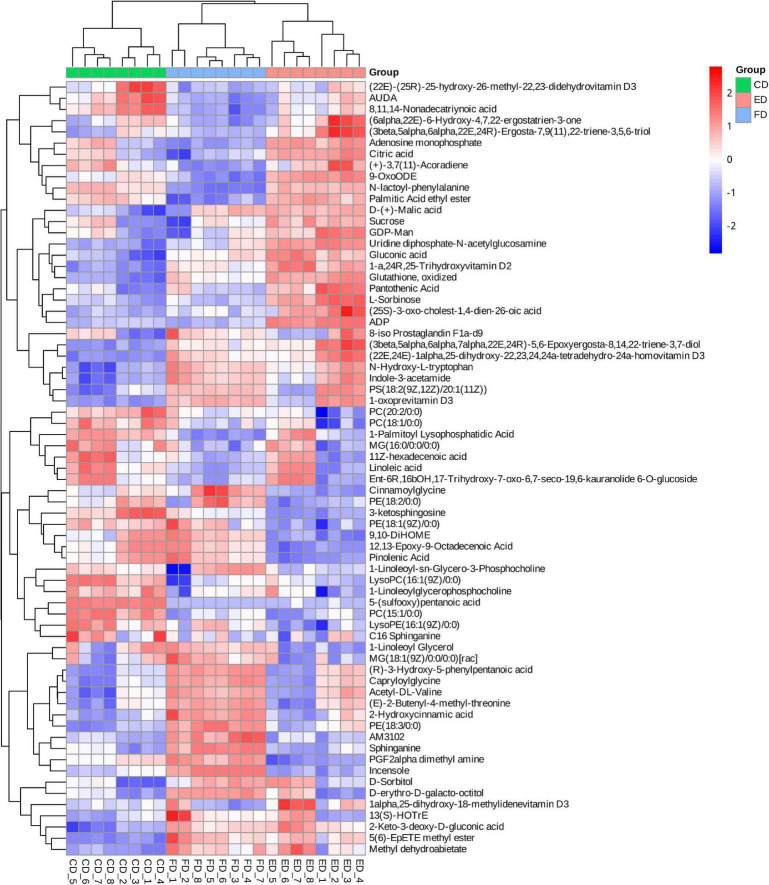
Heatmap analysis of differential metabolite contents among the three drying methods.

The VIP score obtained from the OPLS-DA model could be used to measure the influence intensity and select the most discriminating metabolites. According to the determination criteria of differential metabolites (VIP > 1, *p* < 0.05), a total of 69 differential compounds were filtered from the three drying methods of *D. rubrovolvata* (Table 2), out of which 41 differential metabolites were identified between CD and ED (19 upregulated, 22 downregulated), 53 differential metabolites were observed between CD and FD (23 upregulated, 30 downregulated), and 42 differential metabolites were recorded between FD and ED (17 upregulated, 25 downregulated). Classification and the volcano plot of the differential compounds are shown in Figure 2. All 69 differential metabolites can be categorized into 11 different classes, including glycerolipids (GL, 3), glycerophospholipids (GP, 12), fatty acyls (FA, 16), sphingolipids (SP, 3), sterol lipids (ST, 9), prenol lipids (PR, 3), nucleotides (4), organic acids (3), amino acids (6), carbohydrates (6), and others (4). Among them, lipids, especially FAs and GPs, showed the largest number of differential compounds. To comprehensively show the relationship of different drying methods and the differences in the expression patterns of metabolites, the mass intensities of the differential compounds were determined by hierarchical clustering analysis (Figure 3). The samples from the three groups could be discerned from the heatmap, indicating that the compound profiles were closely related and the drying methods significantly affected the compound types and abundance.

**TABLE 2:** Differential metabolites identification of different drying methods.

No.	Metabolite	m/z	Retention time(s)	Classification	Average peak area		
					ED	CD	FD
1	13(S)-HOTrE/13S-hydroxy-9Z,11E,15Z-octadecatrienoic acid	277.2149	8.460367	Fatty acyls	1770.4228	1273.8719	2481.0104
2	9,10-DiHOME/9,10-dihydroxy-12Z-octadecenoic acid	313.2387	6.527133	Fatty acyls	357.0685	1142.2653	1403.8190
3	9-OxoODE/9-oxo-10,12-octadecadienoic acid	295.2255	8.795017	Fatty acyls	16926.2154	8191.5587	3376.3222
4	AUDA/ 2-(1-adamantylcarbamoylamino)dodecanoic acid	393.3124	13.95545	Fatty acyls	3601.3406	5663.1593	2228.7640
5	Pinolenic acid	279.2304	8.07155	Fatty acyls	4941.4784	19966.0527	22474.3580
6	Linoleic acid	281.2461	13.87495	Fatty acyls	16352.0450	21664.8433	8630.0654
7	12,13-Epoxy-9-Octadecenoic acid	295.2288	8.0679	Fatty acyls	5722.9016	20652.1853	20329.6194
8	8,11,14-Non-adecatriynoic acid	287.1996	13.95545	Fatty acyls	841.7642	1543.1535	444.8693
9	5-(Sulfooxy)pentanoic acid	179.0025	1.20205	Fatty acyls	0.0793	540.4665	0.0067
10	11Z-hexadecenoic acid	293.1775	13.31398	Fatty acyls	6598.8492	8920.1389	4932.9799
11	Palmitic Acid ethyl ester	283.2651	16.82432	Fatty acyls	1395.4597	1286.5839	912.5348
12	5(6)-EpETE methyl ester	333.241	12.37685	Fatty acyls	2103.6776	1268.3266	2422.8153
13	Methyl dehydroabietate	315.2309	15.17222	Fatty acyls	1653.1751	948.3726	1708.8904
14	8-iso Prostaglandin F1a-d9	348.3096	6.55045	Fatty acyls	7270.5599	6655.8042	7864.8306
15	PGF2alpha dimethyl amine	368.3147	7.26975	Fatty acyls	401.7253	1404.2800	2951.5005
16	AM3102/(Z)-(R)-N-((2-Hydroxy-1-methyl)ethyl)-9-octadecenamide	340.3191	8.0134	Fatty acyls	789.1366	759.1938	2107.9510
17	PE(18:2/0:0)	476.2795	7.150267	Glycerophospholipids	3403.2211	4421.2709	4616.1124
18	PE(18:3/0:0)	476.2788	5.53	Glycerophospholipids	159.6071	89.0205	468.0155
19	PE(18:1(9Z)/0:0)	480.306	8.660033	Glycerophospholipids	7002.7463	9936.2410	9823.7474
20	LysoPE(16:1(9Z)/0:0)	452.2752	6.9031	Glycerophospholipids	4124.2980	6016.1834	5233.9906
21	1-Palmitoyl lysophosphatidic acid	409.2342	9.708983	Glycerophospholipids	1170.3098	1633.9476	485.8514
22	PC(20:2/0:0)	548.3686	9.427317	Glycerophospholipids	1894.1768	2608.5643	2189.4709
23	LysoPC(16:1(9Z)/0:0)	538.3166	6.938617	Glycerophospholipids	6260.8600	8656.9195	5825.1069
24	PC(18:1/0:0)	522.3525	8.73735	Glycerophospholipids	33498.9983	45133.7892	35844.9706
25	PC(15:1/0:0)	480.3106	6.371967	Glycerophospholipids	442.4810	868.7591	494.0922
26	1-Linoleoyl-sn-glycero-3-phosphocholine	564.3326	7.188933	Glycerophospholipids	7551.8006	8563.7250	8540.9954
27	1-Linoleoylglycerophosphocholine	520.3369	7.46325	Glycerophospholipids	86848.0771	110545.6924	90430.3593
28	PS(18:2(9Z,12Z)/20:1(11Z))	858.5506	14.5431	Glycerophospholipids	627.5723	258.6269	671.9908
29	3-ketosphingosine	298.2707	6.4523	Sphingolipids	367.3673	2909.2712	937.5769
30	Sphinganine	302.3039	7.873067	Sphingolipids	701.7980	741.4714	1535.5375
31	C16 Sphinganine	274.2729	5.774833	Sphingolipids	7023.1319	8403.4363	7074.5347
32	Ent-6R,16bOH,17-trihydroxy-7-oxo-6,7-seco-19,6-kauranolide 6-O-glucoside	529.2661	9.367833	Prenol lipids	1608.5318	1875.9715	679.2886
33	(+)−3,7(11)-Acoradiene	219.1733	17.2313	Prenol lipids	3265.3768	3029.1563	2424.2188
34	Incensole	307.2619	12.45852	Prenol lipids	11.4040	57.6206	2592.6002
35	MG(18:1(9Z)/0:0/0:0)[rac]	357.2983	14.13527	Glycerolipids	2825.5571	3271.5766	4447.0979
36	MG(16:0/0:0/0:0)	313.2712	13.89528	Glycerolipids	3038.1910	3511.5963	2803.5482
37	1-Linoleoyl glycerol	355.2823	12.45852	Glycerolipids	4303.9384	6396.7161	7335.5620
38	(3beta,5alpha,6alpha,7alpha,22E,24R)-5,6-Epoxyergosta-8,14,22-triene-3,7-diol	427.3183	14.05427	Sterol lipids	2566.6618	928.6939	1683.3784
39	(6alpha,22E)-6-Hydroxy-4,7,22-ergostatrien-3-one	411.3237	15.21188	Sterol lipids	21333.3105	13469.4464	8143.0634
40	(3beta,5alpha,6alpha,22E,24R)-ergosta-7,9(11),22-triene-3,5,6-triol	429.3332	16.418	Sterol lipids	2868.1685	712.1615	496.3490
41	(25S)-3-oxo-cholest-1,4-dien-26-oic acid	427.3186	14.70575	Sterol lipids	3127.9083	1693.6317	1964.5912
42	1alpha,25-dihydroxy-18-methylidenevitamin D3	429.3333	14.39875	Sterol lipids	6118.0662	4221.8496	4222.1627
43	(22E)-(25R)-25-hydroxy-26-methyl-22,23-didehydrovitamin D3	413.3387	15.40822	Sterol lipids	1101.3235	3091.6498	555.4135
44	(22E,24E)-1alpha,25-dihydroxy-22,23,24,24a-tetradehydro-24a-homovitamin D3	427.3165	12.37685	Sterol lipids	1965.4400	927.9248	1673.5349
45	1-oxoprevitamin D3	399.3266	9.526817	Sterol lipids	514.7874	106.2583	577.1674
46	1-a,24R,25-trihydroxyvitamin D2	486.3556	14.86907	Sterol lipids	13696.1722	3447.7737	7530.3405
47	Adenosine monophosphate	348.0698	0.8129	Nucleotides	3187.3452	2128.4282	1298.0226
48	ADP/adenosine diphosphate	426.022	1.076383	Nucleotides	1394.1190	411.4934	385.0216
49	Uridine diphosphate-N-acetylglucosamine	606.0765	0.955733	Nucleotides	6984.5314	3481.2538	4690.8228
50	GDP-Man/guanosine diphosphate mannose	604.0719	1.694533	Nucleotides	1165.8868	643.5380	622.9977
51	Citric acid	191.0203	1.11755	Organic acids	15016.8120	10496.4327	8333.6437
52	(R)-3-Hydroxy-5-phenylpentanoic acid	236.1279	2.894633	Organic acids	1337.7472	734.2479	3379.6802
53	D-(+)-Malic acid	133.0143	0.876067	Organic acids	3632.3160	2212.5281	3125.0811
54	N-lactoyl-phenylalanine	236.0935	2.04935	Amino acids	783.5058	683.5220	120.4064
55	Cinnamoylglycine	188.0698	2.873967	Amino acids	308.6208	610.2346	1240.2244
56	Capryloylglycine	202.143	2.002017	Amino acids	812.6878	439.4036	2270.0014
57	Acetyl-DL-Valine	160.096	0.8524	Amino acids	818.6669	561.4059	1385.4656
58	(E)-2-Butenyl-4-methyl-threonine	188.1271	1.181883	Amino acids	258.0419	186.3875	692.6669
59	N-Hydroxy-L-tryptophan	221.0914	1.5632	Amino acids	3560.5898	1168.7078	4028.3666
60	2-Keto-3-deoxy-D-gluconic acid	177.0409	0.835733	Carbohydrates	1126.0730	649.4276	1077.9559
61	Sucrose	387.1147	0.796567	Carbohydrates	4498.5224	2665.7357	2541.8206
62	L-Sorbinose	179.0565	0.796567	Carbohydrates	4375.2273	2879.3198	3011.8361
63	Gluconic acid	217.0473	0.816067	Carbohydrates	4233.1754	2701.0520	3557.0556
64	D-Sorbitol	181.072	0.796567	Carbohydrates	7395.3677	5533.4971	8084.3991
65	D-erythro-D-galacto-octitol	279.0482	0.816067	Carbohydrates	596.7450	521.5570	956.5947
66	Indole-3-acetamide	175.0861	1.5427	Others	811.4830	312.5419	927.8425
67	Pantothenic acid	218.104	2.425167	Others	12302.4871	9317.4882	9960.2127
68	2-Hydroxycinnamic acid	182.0804	1.161717	Others	8719.1932	7898.6270	13151.1718
69	Glutathione, oxidized	307.0849	1.099383	Others	1649.5317	188.0914	708.1847

**FIGURE 4:**
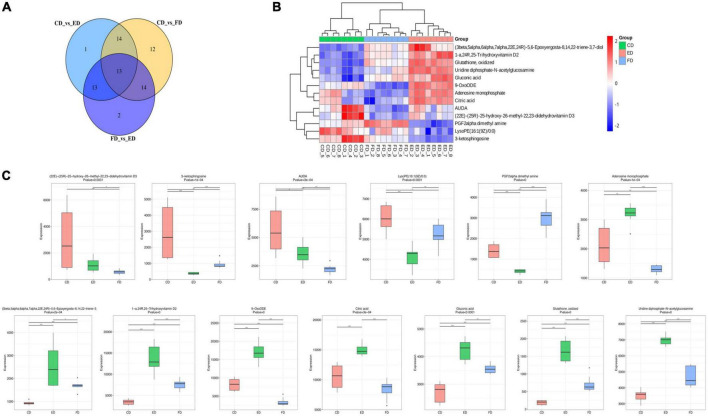
**(A)** Venn diagram of the differential metabolites in different samples; **(B)** heatmap analysis of 13 biomarker contents among the three drying methods; **(C)** boxplots of the normalized peak area of the 13 biomarkers among the three drying methods.

After identifying the differential metabolites with pairwise contrasts, a Venn diagram was used to differentiate the common and exclusive metabolites of *D. rubrovolvata* under different drying methods (Figure 4A). Thirteen metabolites, including eight lipids, two nucleotides, one organic acid, one carbohydrate and one other compound, were mutually defined as compounds with significant difference. These 13 metabolites can be regarded as biomarkers. Their relationships and relative concentrations in the *D. rubrovolvata* samples are shown in the heatmap and boxplots (Figures 4B,C). The levels of most of the biomarkers in the ED and CD samples were significantly higher than those in the FD method, indicating that heating might be a key factor for the difference in metabolites from *D. rubrovolvata*.

### Kyoto Encyclopedia of Genes and Genomes pathway analysis

**FIGURE 5:**
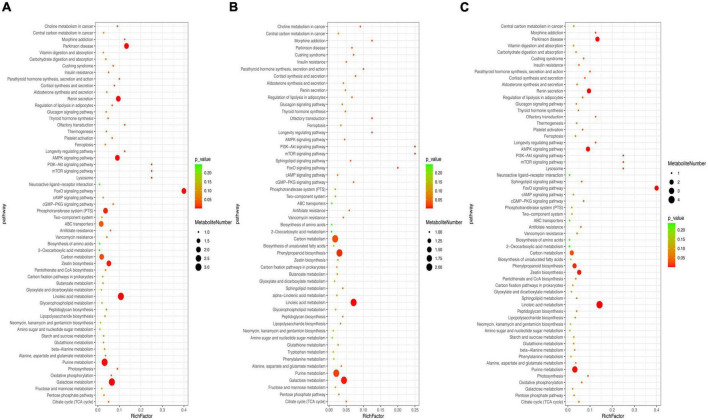
Metabolomic enrichment pathway analysis: **(A)** CD vs. ED, **(B)** CD vs. FD, **(C)** ED vs. FD.

To further explore multiple activities during metabolite accumulation, KEGG enrichment analysis was conducted (Figure 5). The results showed that most of the differential metabolites were assigned to the metabolic pathways and secondary metabolite biosynthesis. The differential metabolites were significantly enriched and mapped into 59, 55, and 60 pathways of the CD vs. ED, CD vs. FD, and FD vs. ED groups, respectively. Despite somewhat distinct rich factors, 9 of the top 20 KEGG pathways were shared in all groups, including galactose metabolism, linoleic acid metabolism, purine metabolism, carbon metabolism, citrate cycle (TCA cycle), pentose phosphate pathway, glutathione metabolism and alanine, aspartate and glutamate metabolism. Intriguingly, these pathways were mainly responsible for the metabolism of active compounds such as lipids, carbohydrates, nucleotides, and amino acids, which was consistent with the observation of the differential metabolites and biomarkers.

### Analysis of differential metabolites and expression according to the drying conditions

**FIGURE 6:**
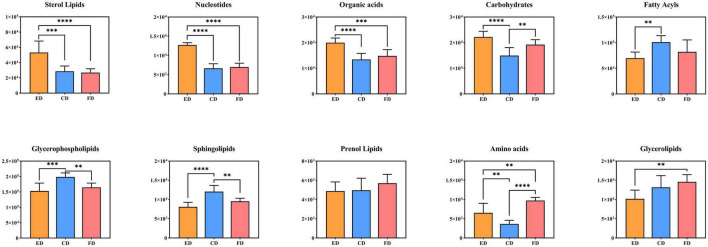
Relative levels of the differential metabolite classes among the three drying methods. ^**^Indicates *p* < 0.01, ^***^indicates *p* < 0.001, and ^****^indicates *p* < 0.0001.

To gain insight into how these metabolites behave in *D. rubrovolvata* treated by different drying methods, the compound abundance of each drying methods was calculated and displayed as bar graphs in Figure 6 for comparative analysis. The results suggested that most of lipids, including FAs, GPs and SPs, had the highest content levels under CD treatment conditions. Furthermore, the STs as well as the nucleotides, organic acids and carbohydrates had the maximum contents by the ED method. In addition, PRs, GLs and amino acids exhibited the highest abundance under FD treatment.

#### Lipids and their derivatives

Lipids are the most abundant compounds of mushroom fruiting bodies and are indispensable to a series of physiological activities because of their flavor, palatability, and nutritive value and are mainly categorized into eight subgroups (18–20). Among the 69 differential metabolites, up to 46 lipids and derivative compounds were screened out in this study, including 16 FAs, 12 GPs, 3 GLs, 3 PRs, 3 SPs, and 9 STs, indicating that drying methods caused a great degree of change in the lipid profile of *D. rubrovolvata*.

It is well-known that FAs are the basic components of lipids, which play an essential role in human health such as regulating cell physiology, reducing cholesterol levels and anti-inflammatory properties (21). In this study, 16 FAs were recognized as the differential metabolites, the majority of which had a significant increase in the total content under the CD treatment. However, these metabolites were scarce under the ED treatment. Among them, fatty acids, especially unsaturated fatty acids, accounted for a great proportion. Mushrooms are great sources of bioactive compounds, food enhancers and functional food products because of their highly beneficial unsaturated fatty acids and other bioactive compounds (22). Additionally, fatty acids easily oxidize or react with other reaction products to form flavoring compounds (23), which contributes to their distinct aroma characteristics. Hence, different drying methods might have a significant impact on the lipids and further affect the quality and flavor of *D. rubrovolvata.*

GPs are essential biomolecules in cells that perform physiological regulatory functions for the human body (24). In this study, GPs were the most abundant differential metabolites in *D. rubrovolvata*, and the total expression showed similar changes as the FAs. The majority of the identified GPs had the highest levels in the CD treatment, while the ED groups had the lowest relative content, indicating that the synthesis of GPs was inhibited or the consumption of GPs was accelerated by the CD treatment. Notably, GPs are fundamental to the cell membrane structure, and the reduction in GPs might cause oxidative stress and result in damage to cells and tissues (25).

SPs are effective physiologically active components and play an essential role in regulating biological processes, especially protecting intestinal cells from inflammatory stress (26–28). In this study, three kinds of SPs showing differential expression were detected, including 3-ketosphingosine, sphinganine, and C16 sphinganine. 3-ketosphingosine known as the first intermediate in the biosynthesis of sphingosine, was upregulated by CD treatment and showed a significant difference in the three drying methods. C16 sphinganine was the most abundant differential sphingolipids in *D. rubrovolvata*. High levels of C16 sphinganine were also found in CD treatment group, and only a slight difference was observed between the ED and FD treatment groups. As a precursor of ceramide and sphingosine, sphinganine showed the highest levels in FD treatment group.

In addition, several other lipids with differential expression were detected in *D. rubrovolvata*. Nine kinds of differential STs were identified, including three ergosterols, five vitamin Ds, and one bile acid. It was possible to observe that the accumulation of STs was not quite consistent in the pattern of FAs, GPs, and SPs. The greatest potential ST contents were found in ED treatment group, followed by CD and FD groups. Ergosterol (previtamin D2), bioactive sterols with anti-inflammatory, antitumor and antimicrobial properties, are the most abundant sterol found in fungal cell membranes (29). The maximum levels of ergosterol were produced in ED treatment group. Hence, the utilization of the ED drying method is a promising approach to obtain ergosterol-enriched dried mushrooms. In addition to ergosterols, five kinds of vitamin D were detected and identified. The trend of vitamin D is in accordance with ergosterol, which also showed upregulation under ED conditions except for (22E)-(25R)-25-hydroxy-26-methyl-22,23-didehydrovitamin D3. For the bile acid (25S)-3-oxo-cholest-1,4-dien-26-oic acid, the greatest potential level was also found in ED treatment group.

In the PRs class, three differential compounds, Ent-6R,16bOH,17-trihydroxy-7-oxo-6,7-seco-19,6-kauranolide 6-O-glucoside, (+)−3,7(11)-accoradiene and incensole, were recognized and classified as terpene glycosides, acorane sesquiterpenoids and diterpenoid compounds. All these are annotated as terpenes, and their total levels followed this trend in rank: FD > CD > ED. It is widely known that terpenes play essential roles in the metabolism of organisms, especially mushrooms, from the components responsible for cell growth to secondary metabolites (30). For the differential metabolites identified in this report, incensole has anti-inflammatory and antidepressant properties due to its ability to activate ion channels in the brain and reduce anxiety or depression, which basically only existed in the FD treatment group. This suggests that high temperatures promote the degradation/transformation of incensole. Sesquiterpenes possess the antibacterial and antitumor activities. As one kind of acorane sesquiterpenoid, (+)-3,7(11)-accoradiene showed little difference in the three drying methods, and the CD treatment showed a slight increase in comparison with the ED and FD treatments.

Three kinds of GLs, namely MG(18:1(9Z)/0:0/0:0)[rac], MG(16:0/0:0/0:0) and 1-linoleoyl glycerol, were also recognized in this study and classified as monoacylglycrols, which are commonly added to commercial food products to mix ingredients. The levels of these components were differentially expressed according to the drying conditions. The total amounts trend in rank was almost consistent with the PRs but without statistically significant differences. Among them, the FD samples stood out for the highest levels of MG(18:1(9Z)/0:0/0:0)[rac] and 1-linoleoyl glycerol, while CD samples stood out for higher concentrations of MG(16:0/0:0/0:0).

#### Amino acids, nucleotides, organic acids, carbohydrates, and their derivatives

The drying method also affects the metabolite concentrations related to flavor and quality (31). To clarify the influence of drying methods on the flavor and quality compounds of *D. rubrovolvata*, we focused on classes of metabolites likely to be major contributors, including amino acids, nucleotides, organic acids, carbohydrates and their derivatives. The heatmap (Figure 3) and the relative levels (Figure 6) presenting these metabolites were differentially expressed depending on the drying method, suggesting that drying methods might influence their taste and quality. The results are described below.

Amino acids have various nutritional and physiological functions in the body and are important flavor components in edible fungi (32). In this study, six differential amino acids and their derivatives were detected, including cinnamoylglycine, capryloylglycine, acetyl-DL-valine, N-lactoyl-phenylalanine, N-hydroxy-L-tryptophan, and (E)-2-butenyl-4-methyl-threonine. The relative levels were increased significantly in the FD treatment group compared to the other two drying methods. Capryloylglycine is frequently used as a conditioning agent or surfactant. Capryloylglycine is a derivative of ferulic acid and is usually used as an antioxidant and food preservative. 5-Hydroxy-l-tryptophan is the precursor in the biosynthesis of 5-hydroxy-tryptamine from l-tryptophan and is usually a popular dietary supplement (33). (E)-2-Butenyl-4-methyl-threonine belongs to the sweet amino acids and their derivatives, while acetyl-DL-valine and N-lactoyl-phenylalanine belong to the bitter amino acids and their derivatives (34). In general, the relative levels of these amino acids and their derivatives in the CD and ED treatment groups were much closer, while the level in FD treatment group was lower.

Nucleotides and their related compounds are key biomolecules in almost all biological processes and are also closely related to the umami taste of mushrooms. Moreover, substantial evidence indicates that nucleotides have essential effects on immune function and maintain optimal physiological function (35). Four different nucleotides and their derivatives were screened in *D. rubrovolvata*, including two nucleotides and two nucleotide sugars. The relative levels of the four nucleotides showed certain differences by different processing methods, and the abundance of the ED treatment group showed an overall upwards trend compared to others. Adenosine monophosphate (AMP) is an important taste nucleotide and has been reported to be a good flavor enhancer to make foods sweeter (36). ADP is involved in a variety of important metabolic and functional regulations, such as promoting vasodilation, preventing thrombosis and vasoconstriction in the liver blood vessels, and increasing hepatic glycogenolysis (37). Uridine diphosphate-N-acetylglucosamine and GDP-Man are two kinds of nucleotide sugars and are well-positioned to serve as glucose sensors. All these metabolites are functional flavor and taste substances and are beneficial to human health. Hence, the variation in these metabolites showed that the macromolecular nucleotides differed greatly after drying and may change the taste and flavor of *D. rubrovolvata*.

In this study, organic acids were also significantly differential metabolites, which were key components in determining the flavor and taste as well as playing a crucial role in the quality and nutritional value (38, 39). Three organic acids were recognized as differential metabolites, including citric acid, D-(+)-malic acid and (R)-3-hydroxy-5-phenylpentanoic acid. Citric acid and malic acid are natural preservatives and are used to add an acidic (sour) taste to foods and carbonated drinks. In our study, the relative levels of citric acid and D-(+)-malic acid in the ED treatment group were higher than those in the other groups, whereas the relative level of (R)-3-hydroxy-5-phenylpentanoic acid was the opposite, and the highest level was observed in the FD treatment group.

Carbohydrates have multiple biological activities and can produce sweetness, which is the main ingredient that determines the taste of edible fungi (40). Here, six differential carbohydrates and their derivatives, including sucrose, D-sorbitol, L-sorbinose, gluconic acid, 2-keto-3-deoxy-D-gluconic acid and D-erythro-D-galacto-octitol, were found. The relative level of the six carbohydrates in ED exceeded those in FD and CD. Among these, two carbohydrates, D-sorbitol and D-erythro-D-galacto-octitol, exhibited increased concentrations under FD conditions.

Altogether, the results indicated that the levels of some characteristic metabolites in *D. rubrovolvata* were significantly affected by the drying methods. During the CD process, aroma character degradation is inevitable, possibly due to direct exposure to air. However, the CD method had the advantage of lipid contents, which might have a non-perishable texture and a low investment cost. The major advantage of the ED method was the controllability of the process parameters, such as the drying temperature and drying time. This was suggested for the preservation of many active substances, such as STs, nucleotides, organic acids and carbohydrates. The FD method showed better preservation for many heat-sensitive and antioxidant activities compounds due to the low temperature and high vacuity. PRs, GLs and amino acids exhibited the highest abundance under FD conditions. Moreover, although this metabolomics profiling strategy can deliver noticeable trends of validity of this methodology, further research is still required to provide insights into the effect of drying methods on quality and flavor by targeted metabolomics approach and others.

## Conclusion

In summary, untargeted metabolomics technology based on UPLC-QTOF-MS was utilized to characterize the effects of drying methods on the metabolic profiles of *D. rubrovolvata.* PCA and OPLS-DA models showed a distinguished separation and identified 69 differential metabolites as well as 13 biomarkers. The differential compounds of the post-dry processing in *D. rubrovolvata* were mainly lipids, amino acids, nucleotides, organic acids, carbohydrates and their derivatives. By comparing the three drying methods, the highest lipid content was attained by CD method. ED method can improve both umami and sweetness and other quality characteristics by retaining more related nucleotides, organic acids and carbohydrates, while the levels of amino acids, PRs and GLs in FD samples were enhanced. Additionally, KEGG enrichment analysis confirmed that pathways pertaining to the biosynthesis of lipids, carbohydrates, nucleotides, and amino acids were significantly induced by drying methods. Taken together, the results of this study shed light on metabolomic profiles and proposed biomarkers of *D. rubrovolvata* subjected to multiple drying approaches. Further studies are warranted to elucidate the underlying molecular mechanisms of these physiochemical alterations.

## Data availability statement

The original contributions presented in this study are included in the article/Supplementary material, further inquiries can be directed to the corresponding author/s.

## Author contributions

HD performed the experiment, generated the data, and wrote the original draft. CZ and XZ conceived and designed the experiments. MC wrote some parts of the article and improved the article further. HG provided the editorial support and polished the grammar. XL, HE, YZ, FZ, TF, ZZ, and HL helped performing the experiment and analyzed the data. All authors contributed to the article and approved the manuscript.

## Footnotes

1
http://www.genome.jp/kegg/pathway.html

## Supplementary material

Supplementary Figure 1 The total ion chromatograms (TICs) *D. rubrovolvata* samples.
