# Supplementary figures and images for: Ultraperformance liquid chromatography-quadrupole time-of-flight mass spectrometry based untargeted metabolomics to reveal the characteristics of Dictyophora rubrovolvata from different drying methods
Source: Front Nutr. 2022 Nov 28;9:1056598. doi: 10.3389/fnut.2022.1056598 (PMC9742599; doi:10.3389/fnut.2022.1056598)

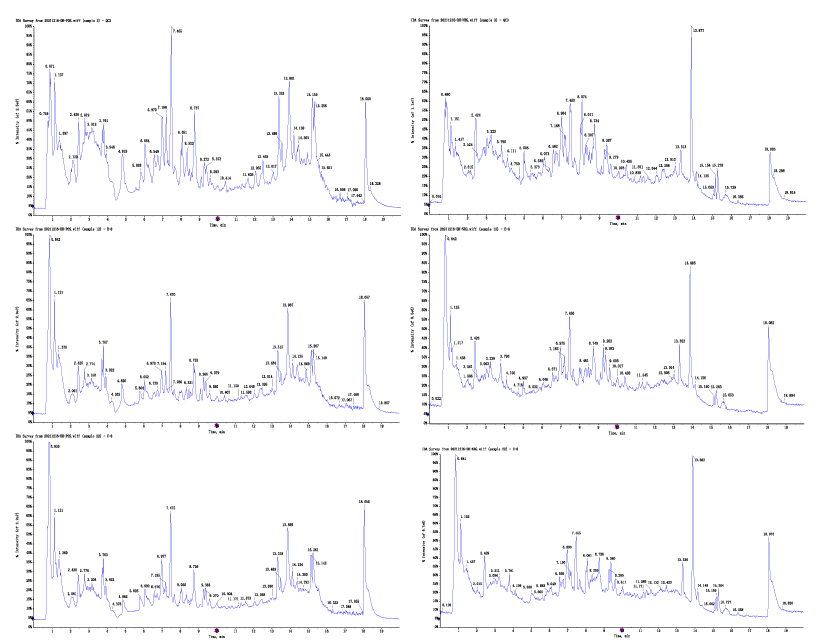

Supplement: Supplementary Figure 1 — The total ion chromatograms (TICs) D. rubrovolvata samples. [file Image_1.tif]
