# Supplementary material for: Ultraperformance liquid chromatography-quadrupole time-of-flight mass spectrometry based untargeted metabolomics to reveal the characteristics of Dictyophora rubrovolvata from different drying methods
Source: Front Nutr. 2022 Nov 28;9:1056598. doi: 10.3389/fnut.2022.1056598 (PMC9742599; doi:10.3389/fnut.2022.1056598)
Supplement: Supplementary file 2 [file Table_1.DOCX]

Table S1. The moisture content of *D. rubrovolvata*

| No. | Group | Moisture content (g/100g) |
| --- | --- | --- |
| C1 | CD | 7.50 |
| C2 |  | 6.97 |
| C3 |  | 7.08 |
| C4 |  | 7.33 |
| C5 |  | 6.79 |
| C6 |  | 7.04 |
| C7 |  | 7.84 |
| C8 |  | 7.51 |
| E1 | ED | 7.96 |
| E2 |  | 7.13 |
| E3 |  | 7.52 |
| E4 |  | 7.61 |
| E5 |  | 6.62 |
| E6 |  | 6.56 |
| E7 |  | 6.32 |
| E8 |  | 6.05 |
| F1 | FD | 7.59 |
| F2 |  | 7.61 |
| F3 |  | 7.85 |
| F4 |  | 7.70 |
| F5 |  | 7.39 |
| F6 |  | 7.28 |
| F7 |  | 7.68 |
| F8 |  | 7.60 |
